# Transcriptional Attenuation Controls Macrolide Inducible Efflux and Resistance in *Streptococcus pneumoniae* and in Other Gram-Positive Bacteria Containing *mef/mel(msr(D))* Elements

**DOI:** 10.1371/journal.pone.0116254

**Published:** 2015-02-19

**Authors:** Scott T. Chancey, Xianhe Bai, Nikhil Kumar, Elliott F. Drabek, Sean C. Daugherty, Thomas Colon, Sandra Ott, Naomi Sengamalay, Lisa Sadzewicz, Luke J. Tallon, Claire M. Fraser, Hervé Tettelin, David S. Stephens

**Affiliations:** 1 Division of Infectious Diseases, Department of Medicine, Emory University School of Medicine, Atlanta, Georgia, United States of America; 2 Laboratories of Microbial Pathogenesis, Department of Veterans Affairs Medical Center, Atlanta, Georgia, United States of America; 3 Institute for Genome Sciences, University of Maryland School of Medicine, Baltimore, Maryland, United States of America; 4 Department of Medicine, University of Maryland School of Medicine, Baltimore, Maryland, United States of America; 5 Department of Microbiology and Immunology, University of Maryland School of Medicine, Baltimore, Maryland, United States of America; University of Kansas Medical Center, UNITED STATES

## Abstract

Macrolide resistance, emerging in *Streptococcus pneumoniae* and other Gram-positive bacteria, is increasingly due to efflux pumps encoded by *mef/mel(msr)* operons found on discrete mobile genetic elements. The regulation of *mef/mel(msr)* in these elements is not well understood. We identified the *mef(E)/mel* transcriptional start, localized the *mef(E)/mel* promoter, and demonstrated attenuation of transcription as a mechanism of regulation of macrolide-inducible *mef*-mediated macrolide resistance in *S*. *pneumoniae*. The *mef(E)/mel* transcriptional start site was a guanine 327 bp upstream of *mef(E)*. Consensus pneumococcal promoter -10 (5′-TATACT-3′) and -35 (5′-TTGAAC-3′) boxes separated by 17 bp were identified 7 bp upstream of the start site. Analysis of the predicted secondary structure of the 327 5’ region identified four pairs of inverted repeats R1-R8 predicted to fold into stem-loops, a small leader peptide [MTASMRLR, (Mef(E)L)] required for macrolide induction and a Rho-independent transcription terminator. RNA-seq analyses provided confirmation of transcriptional attenuation. In addition, expression of *mef(E)L* was also influenced by *mef(E)L*-dependent mRNA stability. The regulatory region 5’ of *mef(E)* was highly conserved in other *mef/mel(msr)*-containing elements including *Tn*1207.1 and the 5612IQ complex in pneumococci and *Tn*1207.3 in Group A streptococci, indicating a regulatory mechanism common to a wide variety of Gram-positive bacteria containing *mef/mel(msr)* elements.

## Introduction

Macrolides are characterized by 14- to 16-membered lactone rings with attached amino sugars which confer an affinity for bacterial ribosomes. Binding of the 23S ribosomal subunit by a macrolide molecule effectively blocks *de novo* synthesis of proteins to inhibit bacterial proliferation. Macrolides are used to treat a wide range of bacterial infections and are particularly important for treatment of infections cause by Gram-positive bacteria. These include, among others, soft tissue infections often caused by *Staphylococcus aureus* and *Streptococcus pyogenes*, and invasive diseases caused by *Streptococcus pneumoniae*. Macrolides are often the drug of choice for empirical treatment of upper respiratory tract infections which are most often caused by *S*. *pneumoniae*. Unfortunately, resistance to macrolides in Gram-positive bacteria, especially in streptococci, complicates the treatment of these diseases. The two major mechanisms of macrolide resistance in these organisms are target modification and active efflux. RNA methylases, such as, Erm(A), Erm(B) and Erm(C) modify specific nucleotides in the 23S rRNA and block macrolide binding. Erm-type methylases confer the MLS_B_ phenotype: high-level resistance to macrolides, lincosamides and streptogramin B. The M phenotype, conferred by macrolide efflux, is generally characterized by lower levels of resistance to 14- and 15-membered macrolides and sensitivity to 16-membered macrolides, lincosamides and streptogramin B.

Macrolide efflux was first described in *Staphylococcus epidermidis* and was attributed to *msr(A)*, an ATP-binding subunit ABC-transporter lacking a membrane-binding domain or an obvious membrane bound partner [1]. Subsequently, the *mef* (macrolide efflux) family of genes encoding major facilitator superfamily-type (MFS) efflux pumps were identified in *S*. *pyogenes* [2] and *S*. *pneumoniae* [3]. Interestingly, *mef* genes are invariably found upstream and are co-transcribed with a homologue of *msr(A)*, named *msr(D)* or *mel*, suggesting a coordinated function between the MFS and the ABC-transporter as a dual efflux pump [4]. Homologues of *mef* and *msr(D)* have been identified in a wide range of Gram-positive bacteria. In *S*. *pneumoniae* efflux is encoded by one of four *mef* alleles: *mef(A)*, *mef(E)*, *mef(O)* or *mef(I)*. The most common in pneumococci is *mef(E)* which is associated with the *msr(D)* homolog *mel* and is located on the 5.5 kb mobile element Mega (Macrolide efflux genetic assembly) [3]. *Mef(A)* is more commonly associated with group A streptococci (GAS) and is usually carried on phage-related elements. In the pneumococcus, *mef(A)* is carried on the degenerative phage element Tn*1207.1* [5]. *mef(I)* was reported in pneumococcal isolates from Spain and is located on the 5612IQ complex, so named because of the presence of the chloramphenicol resistance-encoding *catQ* gene [6].

Macrolide resistance mediated by rRNA methylation or by efflux is inducible by 14- and 15-member ringed macrolides, such as erythromycin and azithromycin, respectively (reviewed in [7]). It is well established that inducible expression of *erm* genes is controlled by attenuation, usually at the level of translation. Transcripts of inducible *erm* genes are characterized by the presence of one or two small (8–20 amino acids) leader peptides encoded 5’ of the *erm* structural gene. Macrolide-bound ribosomes pause at a programmed stall site on the leader peptide disrupting the attenuator and allowing formation of the anti-attenuator. We have previously demonstrated that, unlike most *erm* genes, *mef(E)* and *mel* are induced at the level of transcription of the genes as an operon (*mef(E)/mel*), however the mechanism of induction has not been elucidated [8]. We have also shown that *mef(E)/mel* is induced weakly by the human and mouse antimicrobial peptides LL-37 and CRAMP-38, respectively [8,9]. This has not been shown for *erm* genes. The differences in expression of *mef(E)/mel* and *erm* genes suggest dissimilar mechanisms control these macrolide resistance genes. The goal of this study was to characterize the mechanism controlling macrolide induction of transcription of *mef(E)/mel* by identifying its promoter and searching for *cis*-acting regulatory sequences in the *mef(E)/mel* transcript. In addition, phylogenetically analyses of *mef(E)/mel* and the macrolide efflux systems in other pneumococcal and Gram-positive bacteria.

## Materials and Methods

### Ethics statement

All isolates used in the study were received from previously existing pneumococcal strain collections. Ethics approval was obtained from the Institutional Review Board (IRB) of the institutions who collected the biological specimens from the human subjects: Emory University, Centers for Disease Control and Prevention or Georgia Department of Human Resources. Ethics approval was not required for this study because all data were anonymized.

### Bacterial strains

*Streptococcus pneumoniae* GA17457 is an erythromycin-resistant, Mega-containing, serotype 19A strain originally isolated from the blood of a patient in the Atlanta metropolitan area in 2001 [3, 4]. GA17457 was selected from 147 isolates, including 115 macrolide resistant isolates, most of which were identified through population-based surveillance or carriage studies conducted by the Georgia Emerging Infections Program (GEIP) (S2 Table) [30]. England^14^–9 is an erythromycin resistant, *mef(A)*-containing serotype 14 strain isolated from a patient in the United Kingdom in 1993 [31] (S2 Table). In addition ten strains were isolated from the nasopharynx of healthy children in the Atlanta metropolitan area [32]. Draft genome sequences were deposited into the National Center for Biotechnology (NCBI) Whole Genome Shotgun database (WGS). Isolate metadata and the NCBI accession number for each genome is provided in S2 Table.

Construction of the *mef(E)*-*lacZ* reporter strain XZ7042 from GA17457 has been previously described [8,9]. The control strain XZ7049 was generated by insertion of the promoterless *lacZ* of pPP2 [33] into *bgaA* of GA17457. The Genbank WGS master accession number for GA17457 draft genome sequence is AILS00000000 (S2 Table).

### Construction of mutations in the regulatory region of the *mef(E)-lacZ* reporter

To generate mutations in the *mef(E)/mel* 5’ regulatory region governing *mef(E)-lacZ* expression, plasmid pXZ7032 [9] was used as a template for inverse PCR to introduce point mutations and deletions. For example, the putative *mef(E)/mel* promoter was deleted by amplification of pXZ7032 with overlapping primers 471–777F and 741–777R (S1 Table). The product was *Dpn*I digested and self-ligated to generate plasmid pXB05 which was transformed into GA17457 and selected on 3 μg ml^-1^ tetracycline to generate the XZ7042 *mef(E)-*deletion derivative XB05 (Table 1).

**Table 1:** Strains used in this study.

Strain	Description	References
GA17457	Serotype 19A, ST199 invasive isolate with Mega. Ery^R^	[8,9]
England^14^–9	Serotype 14, ST9 invasive isolate with Tn1207.1. Ery^R^	[31]
XZ7042	GA17457 *bgaA*::*mef(E)*-*lacZ* reporter strain; Ery^R^	[8,9]
XZ7049	GA17457 bgaA::lacZ (promoterless) Mega. Ery^R^	[8]
XZ8009	GA17457 *mef(E)/mel*::*aphA3*. Ery^S^	[9]
XB05	XZ7042 *bgaA*::*mef(E)*Δ-41-(-5)-*lacZ*	This study
XB29	GA17457 *aphA3*-Mega wild type control strain	This study
XB30	GA17457 *aphA3*-MegaΔ+ Δ-41-(-5)	This study
XB30R	XB30 *aphA3-*Mega deletion restored	This study
XB16	XZ7042 *bgaA*::*mef(E)*G+1T-*lacZ*	This study
XB34	GA17457 *aphA3*-MegaΔ+Δ-41-(-5)	This study
XB03	XZ7042 *bgaA*::*mef(E)*Δ+243–280-*lacZ*	This study
XB32	GA17457 *aphA3*-MegaΔ+243–280	This study
XB14	XZ7042 *bgaA*::*mef(E)*Δ+162–189-*lacZ*	This study
XB45	GA17457 *aphA3*-MegaΔ+162–189	This study
XB12	XZ7042 *bgaA*::*mef(E)*Δ+54–92-*lacZ*	This study
XB13	XZ7042 *bgaA*::*mef(E)*Δ+63–80-*lacZ*	This study
XB36	GA17457 *aphA3*-MegaΔ+54–92	This study
XB37	GA17457 *aphA3*-MegaΔ*mef(E)L*-RBS	This study

### Construction of mutations in the regulatory region of the native *mef(E)/mel* operon

To assess the influence of the *mef(E)/mel* 5′ regulatory region on *mef(E)/mel*-mediated macrolide resistance, mutations were introduced into the region in the native locus of GA17457 by allele replacement. Briefly, the kanamycin resistance gene *aphA-3* was fused by overlap extension PCR immediately upstream the 1.1 kb region of Mega upstream of *mef(E)* and cloned into the *Eco*RI site of pSF151 [34] to create plasmid pXB101. GA17457 was transformed with pXB101 and transformants were selected on 400 μg ml^-1^ kanamycin creating XB29 (Table 1) containing the kanamycin-resistance marker immediately upstream of wild type Mega. This inserted the kanamycin resistance marker 780 bp upstream of the *mef(E)/mel* transcriptional start site. Because of the distance between the desired mutation location and the selective marker, recombination events resulting in kanamycin resistance, but without the desired mutation were possible. Therefore, the nucleotide sequence of the entire 1.1 kb region upstream of *mef(E)* of the resulting mutants were determined to confirm the mutations and that no other changes in the regulatory region occurred. Strain XB29 was selected as the positive control strain for erythromycin susceptibility assays.

To generate *mef(E)* regulatory mutants, plasmid pXB101 was used as template for inverse PCR using primers designed to create deletions or introduce point mutations (S1 Table). Inverse PCR products were *Dpn*I digested, ligated and transformed into GA17457 as above. Selection for kanamycin resistance, screening for promoter deletion by PCR and verification of the deletion produced the promoter deletion mutant XB30. XB30 was complemented by restoration of the promoter sequence by transformation of XB30 with a PCR product containing the wild type promoter and selected on 1.0 μg ml^-1^ erythromycin. The sequence of the entire 1.1 kb *mef(E)* regulatory region and the putative promoter deletion were verified by Sanger DNA sequencing.

### Susceptibility assays

The minimum inhibitory concentration (MIC) of erythromycin was determined by Etest as per manufacturer recommendations (AB bioMerieux, Solna, Sweden). To determine the effects of erythromycin induction on resistance, strains were cultured overnight at 37°C in 5% CO_2_ on TSA blood agar plates with or without 10^-1^ MIC concentration of erythromycin representing inducing and non-inducing conditions, respectively. Cell suspensions from the overnight plates were swabbed onto cation-adjusted Mueller-Hinton agar plus 5% horse blood. Plates were overlaid with Etest strips and incubated overnight at 37°C in 5% CO_2_. Erythromycin MIC is reported as μg ml^-1^. Values are the averages of the results of at least three independent experiments. Bold font indicates a greater than 2-fold reduction in erythromycin resistance compared to the similarly treated control XB29. Underlined values indicate at least 3-fold difference compared to the control. ND, not determined

### 5′ RACE

The 5’ end of the *mef(E)/mel* transcript from GA17457 was mapped by 5′ Rapid Amplification of cDNA ends (5′ RACE) as has been previously described [35]. Briefly, total RNA was extracted from GA17457 grown in 10^-1^ MIC erythromycin (12 μg ml^-1^) using the Qiagen RNAeasy Mini Kit (Qiagen Sciences, Germantown, MD). RNA was reverse transcribed into cDNA using Avian Myeloblastosis Virus (AMV) reverse transcriptase (New England Biolabs, Ipswich, MA) and target-specific primers SC10, SC143 and SC199 designed to produce overlapping fragments covering the entire 1.1 kb between the start of the Mega element and the *mef(E)* start codon (S1 Table). Reverse transcription reactions were incubated at 55°C for 1 hr and terminated at 65°C for 10 min. Poly-A tails were added to the 5′ end of cDNAs by incubation with dATP and terminal transferase at 37°C for 20 min. Tailing reactions were terminated at 70°C for 10 min. dA-tailed cDNA was amplified with primer RACE-TT (5′ GTTCAGCGCAGGGTCTTTTTTTTTTTTTT 3′) and the appropriate target-specific primers SC10, SC143 or SC199 (S1 Table). Final amplification was conducted with forward primer RACE-T-F and a nested primer for each reaction (SC10-NEST, SC143-NEST, or SC199-NEST) (S1 Table). Final amplification products were purified and the nucleotide sequence was determined.

### mRNA secondary structure prediction

The secondary structure in RNAFold (ViennaRNA Package 2.0 [11–13]) to

Analyses were performed on the full length e 327 bp region from the promoter to the *mef(E)* start codon and on systematically smaller fragments thereof. In silico, 5’ and 3’ nested deletions and other fragments were tested to account for the effects of 5’ folding during transcription, 5’ to 3’ degradation, and ribosomal interference.

### β-galactosidase assays

To measure expression of *mef(E)*-*lacZ*, cultures of XZ7042 and mutant derivatives were grown to mid-log phase (OD_600_ ~0.3–0.4), divided and exposed to concentrations of erythromycin equal to one-tenth the non-induced MIC of each strain, as determined by Etest (described above). Each subculture was harvested one hour after induction for assessment of β-galactosidase activity. Cultures for time course expression assays were treated as above with cells harvested from continuous cultures at the point of induction and at 30 min intervals thereafter. Expression was determined by standard β-galactosidase protocols [36]. Data was expressed as mean Miller Units ± standard error of the mean (SEM). All experiments were performed in duplicate.

### RNA-Seq

The erythromycin (ERY) minimum inhibitory concentrations were determined by micro-dilution and Etest. Induced MIC was determined in the same manner after overnight growth of the isolates on TSA+ sheep blood agar amended with 10^-1^ the erythromycin MIC of each strain. Mid-log cultures of GA17457 were divided equally into four aliquots and each parallel culture was treated with erythromycin (1.2 μg ml^-1^), LL-37 (200 μg ml^-1^), spiramycin (0.1 μg ml^-1^) or left untreated. Cells were harvested after a one hour incubation and total RNA was extracted, enriched for mRNA, reverse transcribed and sequenced.] Strains were induced at mid-log growth phase (OD_600_) as indicated and cells were harvested after one hour incubation. Total RNA from bacterial samples in each condition was extracted using the QIAGEN RNeasy Mini Kit (Qiagen Sciences, Germantown, MD) followed by DNase treatment in solution and rRNA depletion with the Epicentre Ribo-Zero kit (Epicentre, Madison, WI). Strand-specific RNA-Seq cDNA libraries were constructed and sequenced on the Illumina HiSeq2000 platform; two libraries were combined per channel. The data discussed in this publication have been deposited in NCBI’s Gene Expression Omnibus [37] and are accessible through GEO Series accession number GSE54176 (http://www.ncbi.nlm.nih.gov/geo/query/acc.cgi?acc=GSE54176).

## Results

### Identification of the transcriptional start site of the *mef(E)/mel* operon

**Fig 1:**
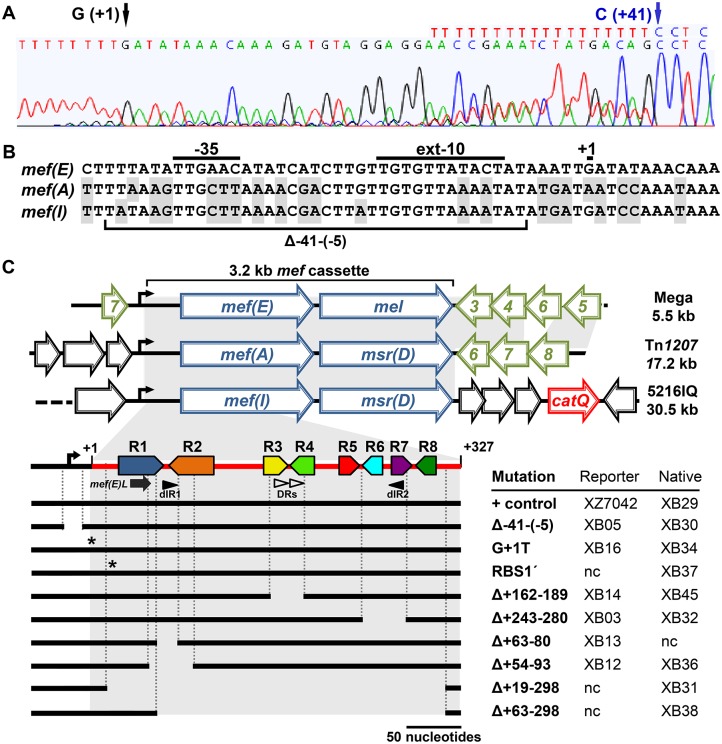
Identification of the promoter for the *mef(E)/mel*(*msr(D)) operon*. **a)** Identification of the operon transcriptional start site in the chromatogram of the nucleotide sequence of 5’ RACE PCR products. Poly-thymine runs indicate the 5’ end of a transcript molecule. Numbered arrows indicate the predicted 5’ termini. **b)** Comparison of the predicted *mef(E)/mel* promoter sequence with putative promoters of other *mef*-containing elements *mef*(A) and *mef*(I) found in *S*. *pneumoniae*. The extended-10 and the-35 promoter sequences are underlined and labeled. The deleted sequence (Δ-41-(-5) of mutations in the native *mef(E)* locus (XB30) or the *mef(E)*-*lacZ r*eporter locus (XB05) is indicated; ‘+1’ indicates the transcriptional start site. Shading indicates dissimilar nucleotides compared to the *mef(E)* promoter region. **c)** The top panel illustrates the genetic organization of *mef*-containing elements in *S*. *pneumoniae*. The homology of the 3.2 “*mef* cassette” was extensive from RBS1 through *mel(msr(D))* as indicated by shading. Blue arrows, macrolide resistance genes; red arrow, chloramphenicol resistance gene *cat*Q; green open arrows, transposon-related genes; open arrows, unrelated genes. The bottom panel illustrates the series of mutations generated in the 5’ regulatory region of the native *mef(E)/mel* locus of GA17457 and the reporter locus, a *mef(E)-lacZ* fusion inserted into *bgaA* in XZ7042. The annotated red line represents the 327 base 5’ *mef(E)/mel* regulatory mRNA region with the *mef(E)/mel* promoter shown as a bent arrow. Each pair of converging, colored solid arrows represents one of four pairs of inverted repeats. Black arrow, *mef(E)L*. closed arrow heads, distal 17 base inverted repeats (dIR1 and dIR2);open arrow heads indicate 12 base direct repeats (DRs). The descriptive name of each mutation and the resulting native-locus and reporter mutant designations are indicated in the right-hand columns; nc, not created.

The serotype 19A invasive pneumococcal isolate GA17457 expresses inducible *mef(E)/mel*-mediated macrolide resistance (Table 1) [8,9]. The start site of the erythromycin-induced *mef(E)/mel* transcript from GA17457 was mapped by 5′ RACE. Unless otherwise stated, all isolates and mutants were induced by one hour exposure to erythromycin at a concentration equal to 10^-1^ minimum inhibitory concentration (MIC). The 5’ RACE reactions produced an approximately 0.4 kb 5′ RACE PCR product. The nucleotide sequence of the 5’ terminus correlated to a guanine residue located 327 bp upstream of *mef(E)* and was tagged with a run of 8–10 consecutive thymine residues which is a hallmark of 5’ RACE (Fig. 1A). Consensus-35 (5′-TTGAAC-3′) and-10 (5′-TATACT-3′) sequences were identified beginning eight base pairs 5′ of guanine transcriptional start (+1G) and were identical to previously identified pneumococcal promoters (Fig. 1B) [10]. The-10 site was also a consensus “extended-10” pneumococcal promoter (5′-TTGTGTTATACT-3′) (Fig. 1B). A second poly-T tract was noted on the 5’ RACE chromatograph (Fig. 1A). The second detected transcript appeared to begin at the cytosine residue (+41C) positioned 287 bases upstream of *mef(E)* (Fig. 1A). However, no promoter sequences were detected immediately upstream of +41C suggesting that it was not an alternate transcription start site. The mRNA molecule terminating with +41C detected by 5’ RACE may be a product of transcript degradation and may reflect biologically relevant endoribonuclease activity (see below).

**Fig 2:**
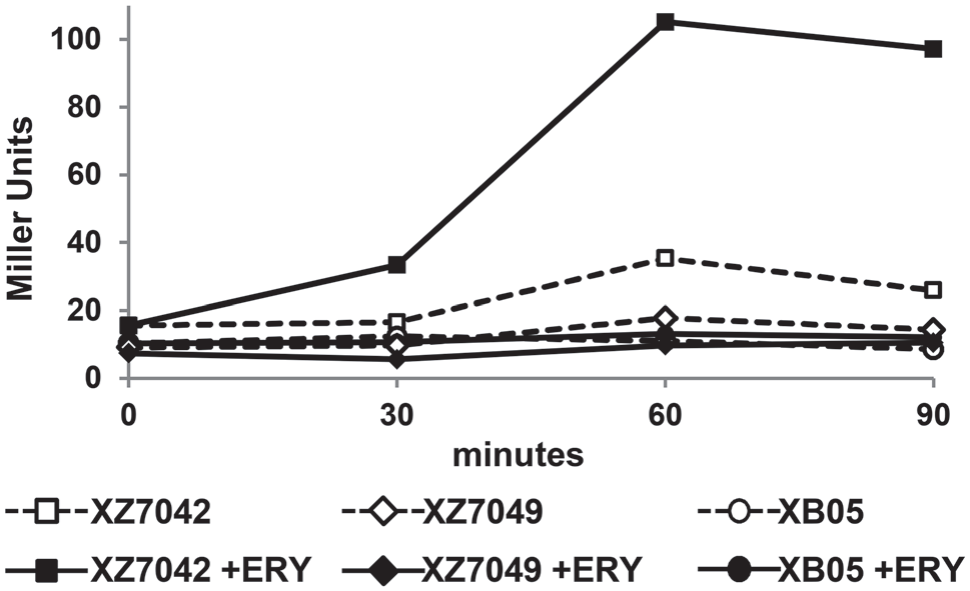
β-galactosidase activity of promoter mutations in the *mef(E)-lacZ* reporter locus. XZ7042 (squares) is the reporter strain generated by insertion of a *mef(E)-lacZ* fusion into the pneumococcal β-galactosidase gene *bgaA*; XZ7049 (diamonds), promoterless *lacZ* strain; XB05 (circles), strain containing the *mef(E)* promoter region deletion (Δ-41-(-5)) in the *mef(E)-lacZ* fusion in the *bgaA* locus. Dashed lines and open symbols, non-inducing conditions; solid lines and closed symbols, inducing conditions (treated with erythromycin at a concentration equal to 10^-1^ erythromycin MIC). Bars represent the standard error of the mean (SEM) of experiments performed in duplicate.

To show that the predicted promoter was required for expression of *mef(E)*, a 37 bp fragment [Δ-41-(-5)] containing the putative-10 and-35 boxes was deleted in the reporter locus inserted into *bgaA* of XZ7042 (Fig. 1B, 1C). The resulting mutant, XB05, was tested for non-induced and erythromycin-induced expression of *mef(E)*-*lacZ* (Fig. 2). As anticipated, the expression of *mef(E)-lacZ* in XZ7042 containing the wild type promoter was induced rapidly by erythromycin and reached maximum induction (6.8-fold) after 1 hour. Expression in the promoter deletion mutant XB05 was negligible, regardless of erythromycin induction and was similar to the negative control, a promoterless *lacZ*, strain XZ7049 (Fig. 2) These data confirm that the promoter was required for transcription of *mef(E)*-*lacZ*. Further, site directed mutagenesis of +1G to a thymine (G+1T) in the reporter locus of the GA17457-derived *mef(E)-lacZ* transcriptional reporter strain XZ7042 resulted in the mutant reporter strain XB16. Under non-inducing conditions β-galactosidase (β-gal) activity of XB16 was not significantly different from XZ7042, however, under erythromycin-inducing conditions, expression by XB16 was 3.9-fold lower, confirming that +1G is required for optimal induction of *mef(E)/mel* (Table 2). To confirm that the promoter was also required for macrolide resistance, the native *mef(E)/mel* locus of GA17457 was introduced by allele replacement using the kanamycin resistance marker *aphA-3* inserted immediately upstream of Mega, strain XB29. Erythromycin resistance in XB29 containing the *aphA-3* and the wild type *mef(E)/mel* regulatory region was identical to the wild type strain GA17457 (MIC 12 μg ml^-1^). Induction of *mef(E)/mel* expression by erythromycin resulted in an erythromycin MIC of 64 μg ml^-1^for XB29, similar to the induced resistance in GA17457 (MIC 48 μg ml^-1^) indicating that the *aphA-3* marker did not influence macrolide resistance or induction. A promoter deletion mutant, XB30, containing the Δ-41-(-5) mutation was sensitive to erythromycin (MIC ≤0.094 μg ml^-1^) under non-inducing and inducing conditions. Restoration of the Δ-41-(-5) deletion in mutant XB30R restored non-induced and induced erythromycin resistance to wild type levels (MIC 8 μg ml^-1^and 64 μg ml^-1^, respectively) (Table 2). Thus, the *mef(E)/mel* transcript contains a 327 base leader sequence including the promoter and also nucleotides not translated as part of the *mef(E)* and *mel* structural genes that be involved in regulation of the operon.

**Table 2:** Influence of regulatory mutations on resistance to erythromycin and expression of ***mef(E)*.**

Mutation^a^	Reporter mutant	*mef(E)-lacZ* expression (Miller units±SEM)^c^		Native locus mutant	Ery MIC (μg ml^-1^)^e^	
		-Ery	+Ery		-Ery	+Ery
**none**	**XZ7042**	13.0±2.9	80.0±4.5	**XB29**	12	64
**-control** ^b^	**XZ7049**	5.1±3.2^d^	7.6±0.2^d^	**XZ8009**	0.019^d^	0.019^d^
**Δ-41-(-5)**	**XB05**	10.5±0.9	16.4±1.9^d^	**XB30**	0.094^d^	0.094^d^
**Δ-41-(-5) C’**	**na**			**XB30R**	8^d^	64
**G+1T**	**XB16**	6.8±1.3	20.4±0.2^d^	**XB34**	4	16^d^
**RBS1’**	**na**			**XB37**	0.125^d^	0.75^d^
**Δ+162–189**	**XB14**	4.8±3.1^d^	11.2±2.5^d^	**XB45**	0.75^d^	0.75^d^
**Δ+243–280**	**XB03**	89.0±0.9^d^	145.7±2.3	**XB32**	48^d^	>256^d^
**Δ+54–93**	**XB12**	8.6±3.1^d^	16.6±2.5	**XB36**	0.12^d^	0.125
**Δ+63–80**	**XB13**	62.2±4.4^d^	64.8±0.9	**na**		
**Δ+19–298**	**na**			**XB31**	12	16^d^
**Δ+63–298**	**na**			**XB38**	32	>256^d^

*a* mutations introduced into the 327 bp regulatory region controlling expression of a *mef(E)-lacZ* transcriptional fusion inserted into the *bgaA* locus of XZ7042 or the regulatory region controlling expression of *mef(E)/mel* in the native Mega locus of GA17457. Numbers are relative to the transcriptional start site (+1). Δ, deletion; C’, mutation complemented by allele replacement; RBS1’, site-direct mutagenesis reverse complementing the *mef(E)L* ribosomal binding site.

*b* negative control. XZ7049, promoterless *mef(E)-lacZ* inserted into *bgaA* of GA17457; XZ8009, *mef(E)/mel* deletion in GA17457.

*c* mean ± standard error of the mean (SEM) β-galactosidase assays performed in duplicate.

*d* indicates a significant difference from the similarly treated control. Relative expression data were analyzed for statistical significance by the unpaired two-tailed Student’s T-test. Changes in MIC values greater or equal to four were considered significant.

*e* MIC, minimum inhibitory concentration, determined by Etest. Values are the mean of at least three independent experiments. MICs are given in micrograms per milliliter. Bold font indicates a greater than 2-fold change in erythromycin resistance compared to the similarly treated control XB29. Underlined values indicate at least 3-fold difference compared to the control. ND, not determined.

### Predictions of the secondary structure of the *mef(E)/mel* 5’ regulatory mRNA

**Fig 3:**
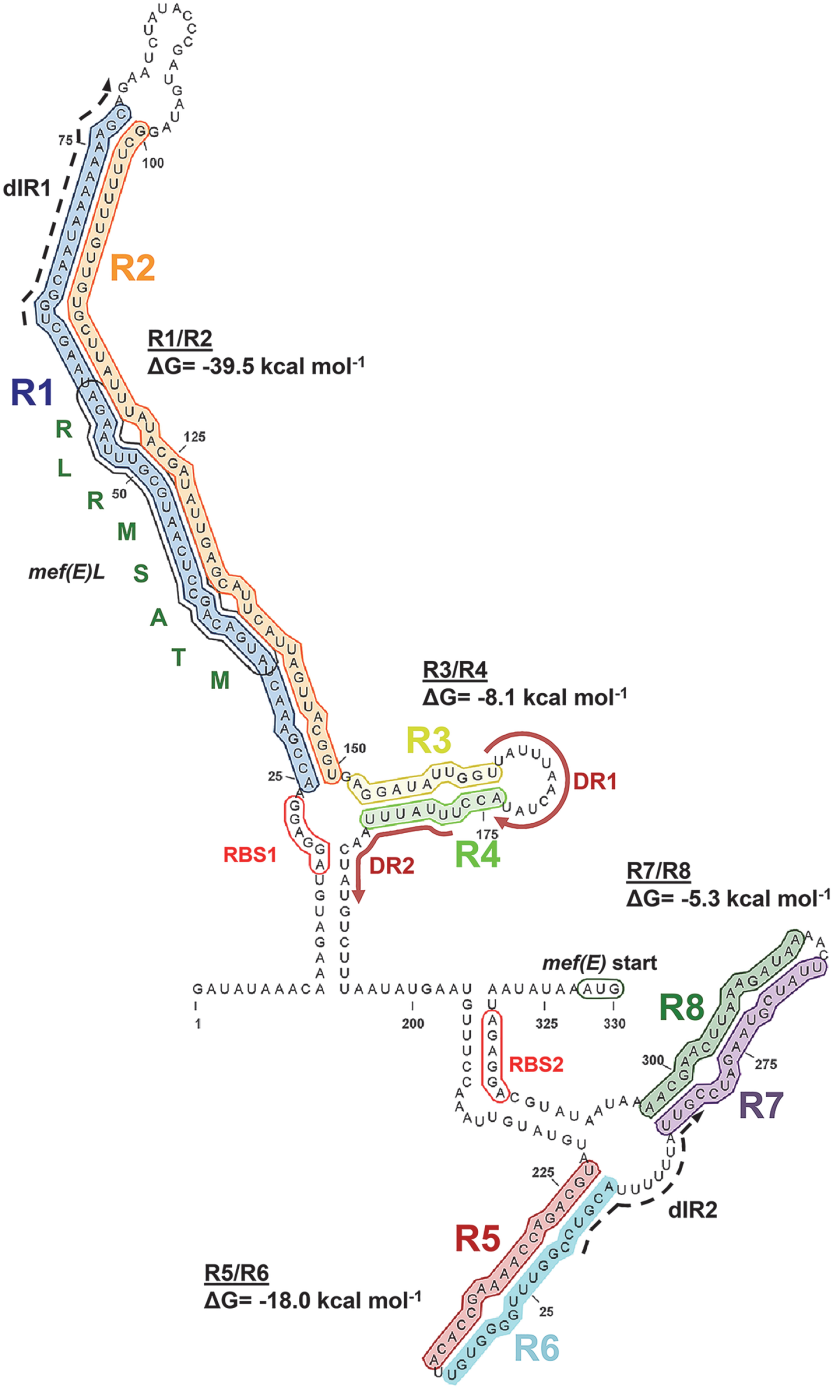
Structure of the *mef(E)/mel* 5′ attenuator structure(transcriptionally inactive). The structure of the 327 nucleotide 5′ mRNA region predicted by RNAFold and visualized with Visualization Applet for RNA (VARNA) [Version 3.8; [43]]. Inverted repeats are shaded with colors consistent with Fig. 1C. The amino acid sequence of the leader peptide Mef(E)L is indicated. The minimum free energy values from each stem-loop predicted by RNAFold analyses of the nucleotides involved in each duplex. Red arrows direct repeats. Dashed arrows, distal inverted repeats; RBS1, *mef(E)L* ribosomal binding site; RBS2 *mef(E)* ribosomal binding site.

Having defined the start of the *mef(E)/mel* transcript and the promoter, we sought to identify *cis*-acting regulatory sequences in the 327 base regulatory leader sequence that may contribute to inducible expression of the *mef(E)/mel* operon. Four pairs of proximal inverted repeats were identified and predicted by RNAFold [11–13]) to form four stem-loops (R1/R2, R3/R4, R5/R6 and R7/R8) (Fig. 1C; Fig. 3). The 54 base repeat R1 annealed to the 51 base R2 to produce a stem-loop (R1/R2) containing a 44 bp stem (37.5% G+C), a 21 base terminal loop. The predicted minimal free energy (ΔG) of the R1/R2 stem-loop was-39.5 kcal mol^-1^ (Fig. 3). Immediately adjacent to R1/R2 was stem-loop R3/R4 containing an eight base pair stem with a ΔG of-8.1 kcal mol^-1^ (Fig. 3). Overlapping the inverted repeats R3 and R4 was a pair of perfect 12 base direct repeats (5′ UUAUUUAACUAU 3′) separated by four bases (red arrows, Fig. 3).

Stem-loop R5/R6, located 39 bp downstream of repeat R4, had the characteristics of a classic Rho-independent transcriptional terminator [14]; the R5/R6 loop was predicted to be stable (ΔG = -18.0 kcal mol^-1^), contained a 55.6% G+C-rich 15 bp stem, and was immediately followed by a run of five uracil residues (Fig. 3). The *erm(K)* transcriptional attenuator has two adjacent Rho-independent transcriptional terminators [15], raising the possibility that last stem-loop R7/R8 in the *mef(E)/mel* 5′ regulatory mRNA was a second terminator. However, R7/R8 did not have the characteristics of a Rho-independent terminator; the stem was not G+C-rich (14.3%), it was not predicted to be thermodynamically stable (ΔG-5.3 kcal mol^-1^) and was not followed by a poly-uracil tract.

### Identification of a leader peptide *mef(E)L* required for *mef(E)/mel* expression

Expression of inducible *erm* methylase genes occurs when erythromycin-bound ribosomes stall during translation of a small leader peptide encoded upstream of the structural gene on the *erm* transcript. A search of the 327 base 5’ regulatory sequence of the *mef(E)/mel* transcript revealed a small (eight codon) open reading frame (hereafter called *mef(E)L*) located 34 bases from +1G and preceded by a consensus ribosomal binding site (5’ AGGAGG 3’) (hereafter called RBS1) (Fig. 1C; Fig. 3). The peptide sequence of Mef(E)L (MTASRLR) was similar to predicted and confirmed leader peptides of other macrolide resistance determinants (Table 3). Notably, Mef(E)L was homologous to the *Bacillus lichenifornis erm(K)* leader peptide Erm(K)L (MTHSMRLRFPTLNQ) and the putative leader peptide Msr(A)L (MTASMRLR) encoded upstream of the macrolide-inducible *mel(msr(D))* homolog *msr(A)* from *S*. *epidermidis* (Table 3) [1,15]. The similarity of *mef(E)L* to *erm(K)* was of note because *erm(K)* is one of the few *erm*-type methylase genes known to be regulated by transcriptional attenuation [15] and *mef(E)/mel* is transcriptionally regulated [4,8]. The minimal leader peptide sequence required for erythromycin induction of *erm(K)* has been shown to be MTHSMRLR [15,16], nearly identical to Mef(E)L suggesting that the *mef(E)* leader peptide may be the site of ribosome stalling in the presence of macrolides. Apart from the similarities of the leader peptide, no discernible homology was noted between the 327 base *mef(E)/mel* 5′ regulatory region and the 354 base 5′ regulatory region of *erm(K*).

**Table 3:** Putative leader peptide sequences of inducible macrolide resistance genes.

Structural gene	Peptide sequence^a^	Species	Genbank accession no. (reference)
*mef(E)*	MTASMRLR	*S*. *pneumoniae*	AILS01000012 (This study)
*mef(A)*	MTASMRLR	*S*. *pyogenes*	AY657002 [38]
*mef(A)*	MTASMRLR	*S*. *pneumoniae*	AILI00000000 (This study)
*mef(I)*	MTASMRLR	*S*. *pneumoniae*	AJ971089 [6]
*mef variant 1*	MTASMRLR	*S*. *suis*	CP002465 [39]
*msr(A)*	MTASMRLK	*Staphylococcus epidermidis*	X52085 [1]
*msr(C)*	MTASMKLRFELLNNN	*Enterococcus*	ABD51781 [40]
*erm(K)*	MTHAMRLRFPTLNQ	*B*. *licheniformis*	M77505 [41]
*erm(D)*	MTHSMRLR	*B*. *licheniformis*	M29832 [42]
*ermJ*	MTHSMRLRFPTLN	*B*. *anthracis*	L08389 [13]

^a^ underlined sequence represents amino acid residues conserved in the programmed ribosome stall site of *erm(K)* [16].

To confirm that translation of the predicted leader peptide *mef(E)L* was required for *mef(E)/mel* expression, the native locus *mef(E)L* was translationally inactivated by altering the ribosomal binding site from AGGAGG (RBS1) to CCTCCT (RBS1’) in the mutant XB37 (Fig. 1C; Table 2). XB37 was sensitive to erythromycin in inducing and non-inducing conditions indicating that *mef(E)L* expression was required for *mef(E)*/*mel*-mediated resistance (Table 2). These data were consistent with transcriptional attenuation as a mechanism whereby stalled macrolide-bound ribosomes interfere with stem-loop R1/R2 to allow the formation of an anti-attenuator.

### Stem-loop R5/R6 is a Rho-independent transcriptional terminator

The stem-loop structure R5/R6 was present in the predicted attenuator but absent from the predicted anti-attenuator structure consistent with the conclusion that R5/R6 represents a Rho-independent transcriptional terminator in non-inducing conditions. To determine if R5/R6 was required for repression of *mef(E)/mel* expression in non-inducing conditions a 39 bp deletion (Δ+243–280) containing repeat R6 and the 5′ end of R7 was introduced into the reporter locus of XZ7042 and into the native Mega locus of GA17457 creating XB03 and XB32, respectively (Fig. 1C). The non-induced expression of *mef(E)-lacZ* in XB03 (89.0 m.u.) was 6.8-fold higher than non-induced expression in XZ7042 and was nearly the same as induced expression in XZ7042 (Table 2). However, expression in the XB03 remained inducible, increasing 1.6-fold to 145.7 m.u. after induction by erythromycin (Table 2). This may suggest an additional mechanism of regulation and induction.

Erythromycin resistance of XB32, the native locus ∆+243–280 mutant, in non-inducing conditions was 4-fold higher than in the native locus control XB29 (Table 2). Erythromycin resistance in macrolide inducing conditions exceeded the limit of the Etest assay (MIC>256 μg ml^-1^), >4x higher than XB29 (MIC 48 μg ml^-1^) (Table 2). These data further indicated that stem-loop R5/R6 was needed for repression of *mef(E)/mel* transcription, and that R5/R6 functioned as a Rho-independent transcriptional terminator.

### Prediction of the anti-attenuator structure of the *mef(E)/mel* transcript

To identify the anti-attenuator structure, additional inverted repeats in the 327 base leader sequence that could be involved in alternate folding of the *mef(E)/mel* transcript were identified. A pair adenine-uracil rich 18 base repeats separated by 174 bases, predicted not to anneal were located in the attenuator structure (dashed arrows, Fig. 3). The first unit of these distal repeats, dIR1, was located at the 3’ end of R1, six bases downstream of *mef(E)L* suggesting availability for pairing when a stalled ribosome disrupts the R1/R2 stem-loop. The second distal repeat unit, dIR2 overlapped the Rho-independent transcriptional terminator and included a poly-U tract such that pairing of the distal inverted repeats dIR1 and dIR2 would disrupt the transcriptional terminator and allow transcription to proceed. Under the presumption that ribosome stalling would prevent folding of the transcript from the start through the *mef(E)L* coding sequence, the 5′ terminal 63 bases were excluded from interacting in the *in silico* folding of the regulatory region

**Fig 4:**
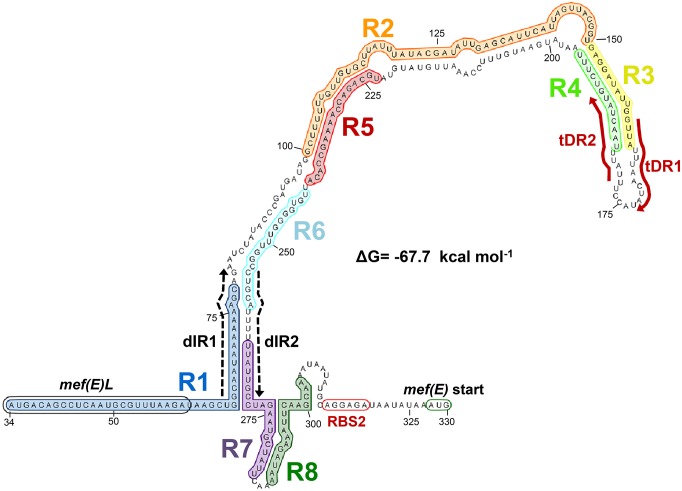
Structure of the *mef(E)/mel* 5′ anti-attenuator structure (transcriptionally active). Structure generated by RNAFold of the 249 bases remaining after removing of the 64 nucleotides from the 5′ terminus predicted to be unavailable for base paring due to macrolide-induced ribosomal stalling. Annotations are the same as stated for Fig. 1C.

The predicted transcriptionally active anti-attenuator structure is shown in Fig. 4. The 5′ terminal 63 bases were excluded from interacting in the *in silico* folding of the regulatory region under the presumption that ribosome stalling would prevent folding of the transcript from the start through the *mef(E)L* coding sequence. The leader sequence +60-+327 was predicted to fold into a stable (ΔG-67 kcal mol^-1^) containing a duplex formed by annealing of dIR1 and dIR2 (Fig. 4). Significantly, the putative Rho-independent terminator R5/R6 was resolved, supporting the predicted identity of the anti-attenuator structure (Fig. 4).

**Fig 5:**
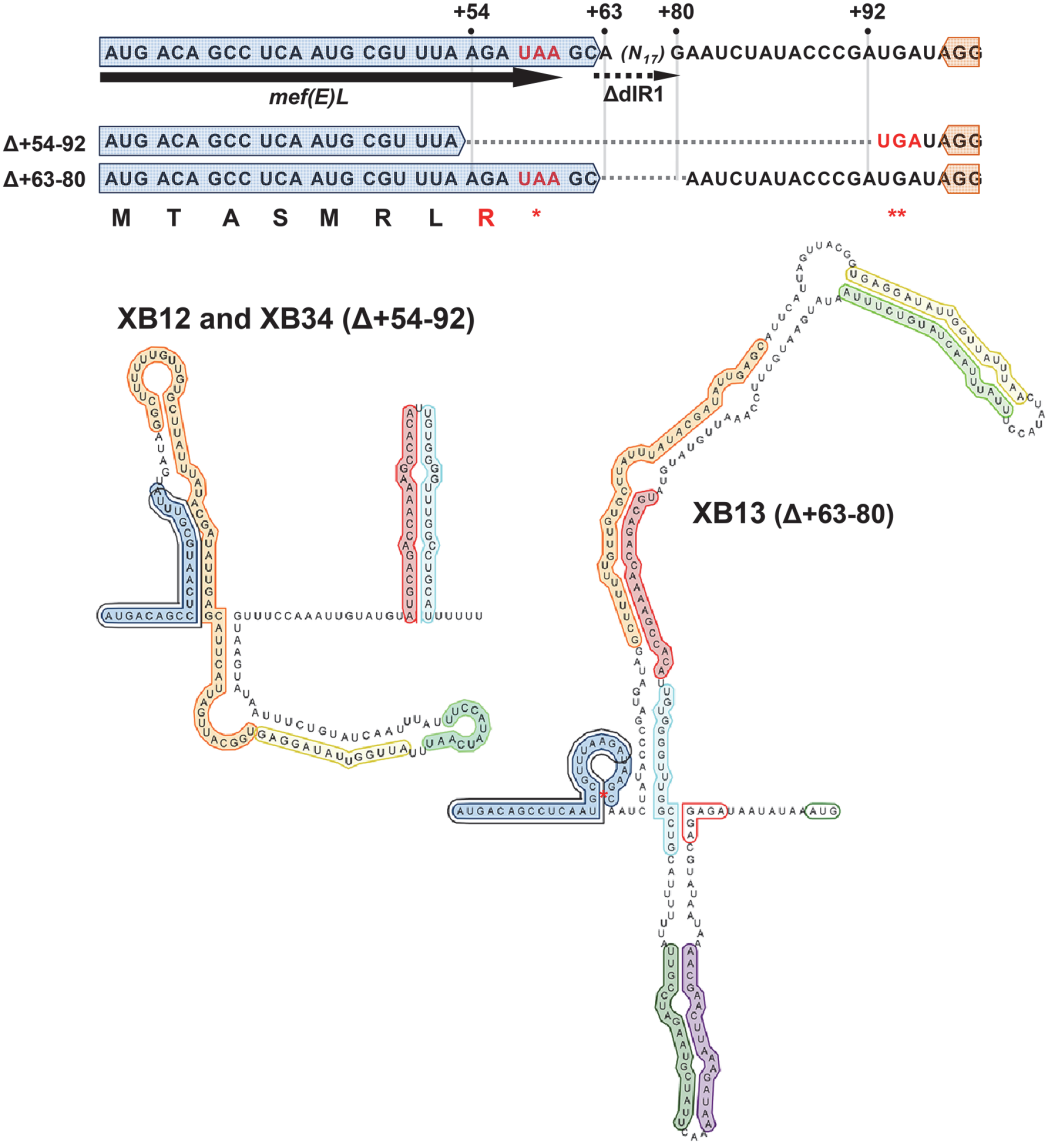
Stem-loop R1/R2 and *mef(E)L*. The sequence and predicted structure of the 5’ end of *mef(E)/mel* transcripts with the Δ+54–92 and Δ+63–80 mutations. Nucleotides are numbered relative to the *mef(E)/mel* transcriptional start (+1). The inverted repeats are annotated as described for Fig. 3. A single asterisk signifies a stop codon of the full-length *mef(E)L*. Double asterisks indicate the stop codon generates by the Δ+54–92 mutation. The dashed arrow indicates the location of the first unit of the distal inverted repeats (dIR1).

To experimentally test the anti-attenuator structure, a deletion of 18 bases corresponding to dIR1 (Δ+63–80) was introduced into the reporter locus of XZ7042 creating XB13 (Fig. 1C). Folding analyses predicted deletion of dIR1 would destabilized the R1/R2 stem-loop, result in the annealing of R6 and the novel sequence created between the truncated R1 and the repeat R2 (Fig. 5), and prevent the formation of the R5/R6 terminator. This favored formation of the anti-attenuator structure even under non-inducing conditions (Fig. 5). The model of attenuation due to a single Rho-independent transcriptional terminator would predict this mutant to constitutively expression *mef(E)/mel* and macrolide resistance (Fig. 5). Indeed, in non-inducing conditions, expression of *mef(E)-lacZ* was 4.8 times higher in XB13 (62.2 m.u.) than in the wild-type reporter XZ7042 (13.0 m.u.), and was not further induced by erythromycin exposure (64.8 m.u.) (Table 2).

The roles of stem-loop R1/R2 and the leader peptide *mef(E)L* were further tested by introduction of a second deletion (Δ+54–92) into the reporter locus to create the reporter mutant strain XB12 (Fig. 1C). Like Δ+63–80, this mutation deleted dIR1 but extended upstream to disrupt *mef(E)L*. Folding analyses of the Δ+54–92 deletion predicted that the transcriptional terminator R5/R6 would form in XB12 (Fig. 5) and would form a R1/R2 stem-loop, albeit with reduced stability. The Δ+54–92 deletion also resulted in a truncation of *mef(E)L* due to substitution of the arginine codon (AGA) in the eighth position with an opal stop codon (UGA) (Fig. 5). The premature termination of the leader peptide was predicted to prevent ribosome stalling and thus render *mef(E)/mel* expression uninducible.

Indeed, basal expression of β-gal activity in the Δ+54–92 reporter mutant XB12 was not significantly different from the negative controls XZ7049 or XB05 and, under inducing conditions expression was 4.8-fold lower than wild-type (16.6 m.u. and 80.0 m.u., respectively) indicating a nearly complete loss of induction by macrolides (Table 2). Consistent with this result, the Δ+54–92 native locus mutant XB36 (Fig. 1C) was susceptible to erythromycin regardless of prior erythromycin induction (MICs 0.125 μg ml^-1^) (Table 2). These data confirm the role of stem-loop R1/R2, and the leader peptide *mef(E)L*, and support the theory that induction of *mef(E)/mel* is due to ribosome stalling at *mef(E)L* in the presence of inducing macrolides.

### Stem-loop R3/R4 is also required for the regulation of *mef(E)/mel* expression

**Fig 6:**
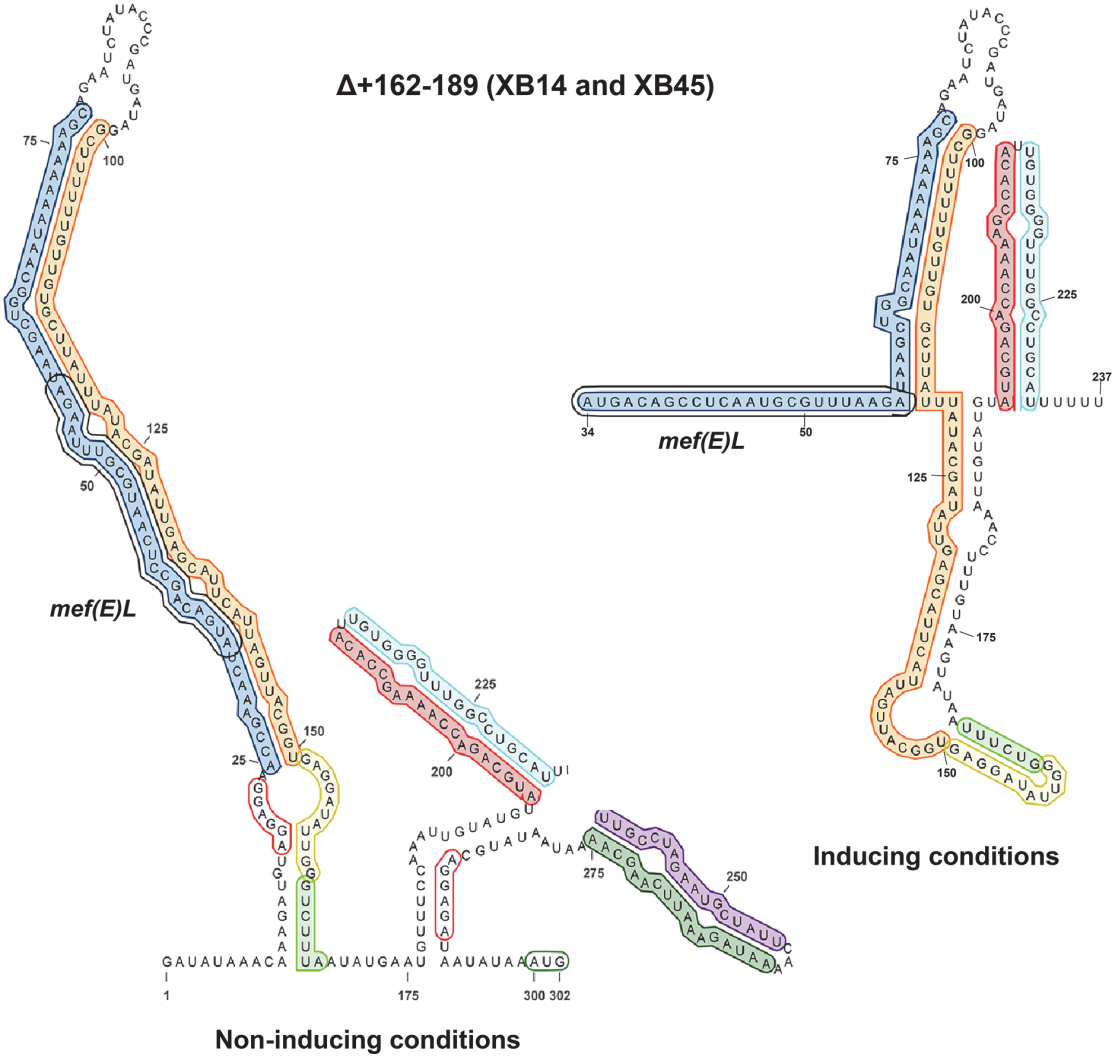
Stem-loop R3/R4 is required for *mef(E)/mel* expression. The predicted secondary structures of the Δ+162–189 *mef(E)/mel* regulatory region in (a) non-inducing and (b) inducing conditions. Disruption of R3/R4 by mutation was predicted to not disrupt the R5/R6 transcriptional terminator in inducing or non-inducing conditions, indicating that mutants with the Δ+162–189 deletion were uninducible. Annotations are consistent with those described in the Fig. 1C legend. Inducing conditions include growth with exposure to erythromycin at a concentration equal to 10^-1^ the erythromycin MIC.

Stem-loop R3/R4 was formed by imperfect annealing of R3 and R4 repeats (Fig. 3). R3/R4 contained a pair of 12 base direct repeats (Fig. 3, red arrows) and could function as a *cis*-acting operator, influencing *mef(E)/mel* expression through interaction with *trans*-acting regulatory factors. To determine if R3/R4 influenced *mef(E)/mel* expression, 28 bp containing the direct repeats (Δ+162–189) was deleted from the *mef(E)-lacZ* reporter locus to create reporter strain XB14 (Fig. 1C). Folding analyses predicted the terminator would form even without erythromycin induction (Fig. 6). β-gal activity expressed in XB14 grown with or without erythromycin induction was markedly reduced and was similar to the negative controls XZ7049 or XB05, demonstrating that *mef(E)-lacZ* was not expressed in an R3/R4 mutant (Table 2). Likewise, XB45 containing the Δ+162–189 deletion in Mega was susceptible to erythromycin with or without induction with erythromycin (MICs 0.75 μg ml^-1^) (Table 2). These data confirmed the requirement of stem-loop R3/R4 in the regulation of *mef(*E*)/mel* expression.

### Mef(E)/Mel regulation differs from the attenuation model of *erm* methylases

The observation that the R5/R6 terminator deletion mutants remained inducible to macrolides suggested the presence of unidentified secondary structures or an additional level of regulation. Deletion of the 5’ leader sequence, or disruption of the 5’ secondary structures, leads to constitutive expression or *erm* methylases such as *erm(A)*, *erm(B)* and *erm(C)* [17,18]. To assess additional levels of regulation, bases +19 to +298 were deleted from the native *mef(E)/mel* locus and erythromycin resistance was determined. Erythromycin resistance of non-induced the Δ+19–298 mutant, XB31 (Fig. 1C), was not different from the non-induced control strain XB29 (MIC, 12 μg ml^-1^) (Table 2). However, resistance was not induced significantly by erythromycin induction (MIC, 16 μg ml^-1^) which represented a significant change from the induced control (MIC, 64 μg ml^-1^) (Table 2). This result was also not consistent with the simple attenuation model observed with *erm* methylases.

Ribosomal stalling protects nascent transcripts from degradation by ribonucleases [19]. The deletion of XB31 removed *mef(E)L* and thus ribosome stalling was not possible, suggesting that the XB31 *mef(E)/mel* transcript, though not attenuated, was unstable and quickly degraded, preventing over-expression of the efflux pump. To test this hypothesis, mutant XB38 was generated by deletion of bases +63–298. The mutation was identical to that found in XB31 except that the leader peptide and the putative ribosome stall site remained (Fig. 1C). The non-induced resistance of XB38 to erythromycin (MIC, 32 μg ml^-1^) was 2.7-fold higher than the control XB29, and at least 4-fold higher after exposure to erythromycin (MIC, >264 μg ml^-1^) (Table 2). These data indicated that the *mef(E)/mel* transcript was protected from degradation by ribosomes stalled during translation of the leader peptide. Further, 5’ RACE detected transcripts that begin with the +41C (Fig. 1A), located in the third codon of *mef(E)L*, or five codons from its 3’ end (see above). This was consistent with the reported endonuclease cleavage site of the *erm(C)* leader peptide *erm(C)L* which was shown to be protected by the stalled ribosome [19]. The data suggest that *mef(E)L* translating ribosomes stall on the last codon of *mef(E)L* and protect the transcript from further degradation.

### Visualization of *mef(E)/mel* attenuation by RNA-seq

**Fig 7:**
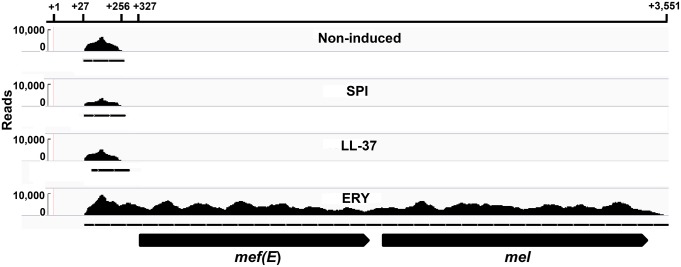
Visualization of *mef(E)* attenuator by RNA-Seq. Whole transcriptome analysis by RNA-Seq of mRNA from the wild type strain GA17457, non- induced or exposed to spiramycin, the antimicrobial peptide, LL-37, or erythromycin. The number of sequence reads correlated to the *mef(E)/mel* transcript is shown on the graph. Dashed lines below each sample indicate transcripts predicted by Integrated Genome Viewer (IGV). The *mef(E)* and *mel* open reading frames are shown below.

RNA-Seq whole transcriptome analyses were utilized to further determine if the *mef(E)/mel* transcript was terminated prematurely in non-inducing conditions. Fig. 7 shows the reads mapping to the *mef(E)/mel* operon viewed using the Integrative Genomics Viewer (IGV) (Broad Institute, Cambridge, MA). The tracks represent expression on the forward strand (reverse strand expression data not shown) under each experimental treatment. As expected, the *mef(E)/mel* operon was not expressed in untreated samples or in samples treated with the non-inducing macrolide spiramycin (Fig. 7). LL-37, a weak inducer of *mef(E)/mel* [9], also did not induce *mef(E)/mel* under these conditions. In the erythromycin-induced sample, *mef(E)* and *mel* were expressed as a single operon (Fig. 7). Interestingly, in the non-induced samples, a low level of expression was observed originating downstream of the transcriptional start site and terminating after approximately 250 bases. In the erythromycin-induced sample, transcription began at the +1G and continued until the end of the *mef(E)/mel* operon (Fig. 7). Similar expression patterns for *mefE/mel* expression (non-induced or induced with erythromycin) were observed in whole transcriptome analyses of three other Mega-containing invasive pneumococcal isolates, GA17545 (AFGA00000000.1), GA02254 (AIKI00000000.1) and GA41565 (AGPO00000000.1) (data not shown). RNA-Seq demonstrated transcriptional attenuation of *mef(E)/mel* in non-inducing conditions and transcriptional activation in the presence of the inducing macrolide.

### Phylogenetic analyses of *mef*-containing mobile elements

As part of a comparative genomic study of the evolution and dissemination of antimicrobial resistance in a geographically defined population of *S*. *pneumoniae*, the genomes of 147 pneumococcal isolates, including 115 macrolide resistant invasive pneumococcal strains, were sequenced. Isolate metadata and the NCBI accession number for each genome are provided in S2 Table. Mega carrying *mef(E)* was present in 102 macrolide resistant isolates, including 20 that also contained *erm(B*) carried on Tn*2010*. Strain England^14^–9 (accession no. NZ_AILI00000000 contained *mef(A)/msr(D)* located on the mobile element Tn*1207.1*. All other isolates contained either *erm(B)* alone (n = 11) or 23S ribosomal mutations (n = 1). Type I Mega (5.5 kb) was found in 61 isolates, 49 isolates had the type 2 Mega (5.4 kb) [3] and a single isolate contained a 112 bp duplication of the 5′ leader sequence of *mel* that has not been previously described (data not shown). Of the 102 Mega sequences, 77 were identical and the remaining contained no more than three single nucleotide polymorphisms (SNPs). The exception was the original Mega sequence (AJ274302), which contained a 16 bp insertion that has been described in *S*. *viridians* (EF042094) and many *mef*-containing elements from non-pneumococcal streptococci.

Mef(E)/Mel-mediated resistance to erythromycin varied from 1 μg ml^-1^ to >64 μg ml^-1^ in the 102 Mega strains. However, phylogenetic analyses of the Mega sequence revealed no changes within the *mef(E)* or *mel* structural genes that correlated with the strain to strain variation in macrolide resistance, suggesting that variable resistance levels were due to differential expression of *mef(E)/mel*. Deletions in the *mef(E)/mel* 5′ regulatory region have not been reported and none were detected in the 102 Mega-containing isolates examined in this study.

Alignment of Mega with pneumococcal elements containing *mef(A)* (*Tn*1207.1) and *mef(I)* (5216IQ complex) using Clustal Omega (version 1.2.1) [20] revealed a 3.2 kb region of homology, “the *mef* cassette” including the 5′ regulatory region of *mef(E)* and *mel* (Fig. 1A). The *mef* cassette contained the-10 consensus sequence at its left junction and was inserted in all of the elements just downstream of the-35 box of the *mef/mel(msr(D))* operons resulting in evolutionary divergence of the-10 and-35 promoter sequences (Fig. 1A). The promoters displayed some nucleotide similarity (approximately 65% identity), but the 327 bp 5′ regions of the *mef(A)* and *mef(I)* elements were 96% and 97% identical, respectively, to that found in *mef(E)* (Fig. 1A). These data suggest an evolutionary history of inter- and intra-species horizontal transfer of the 3.2 Kb *mef* cassette.

A consensus attenuator structure was generated by Clustal Omega alignment of the 5′ regulatory mRNA region of these other *mef*-containing elements and prediction of consensus secondary structures performed by RNAlifold (ViennaRNA Package 2.0 [11]). The *mef*-containing elements included the pneumococcal *mef(I)-* and *mef(A)*-containing elements (5216IQ complex and *Tn*1207.1, respectively), and elements from *S*. *pyogenes*, *Clostridium kluyverii*, *C*. *perfringens* and Group G streptococci. The consensus structure was not significantly different from the *mef(E)/mel* attenuator structure (Fig. 3).

**Fig 8:**
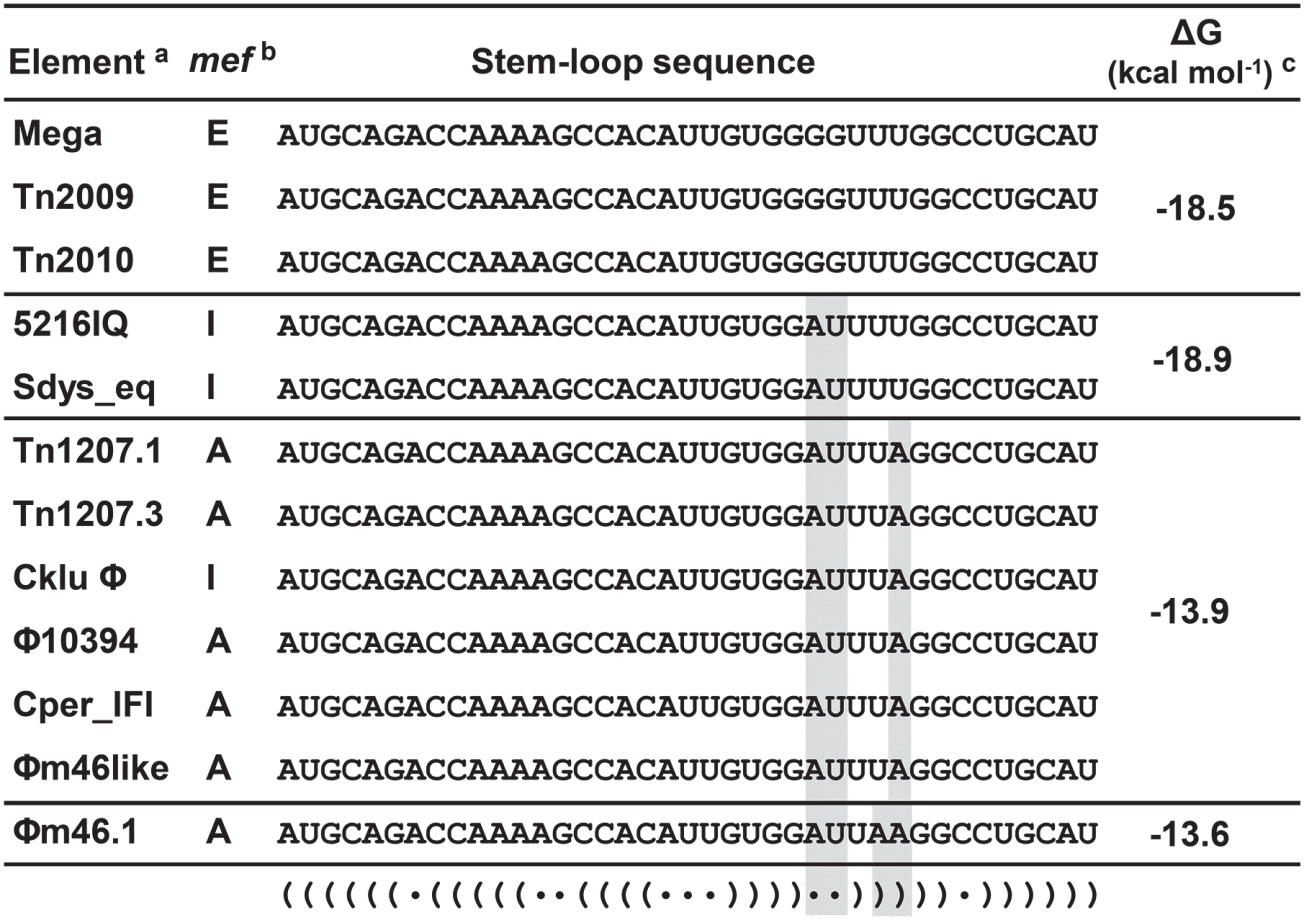
Comparison of the Rho-independent terminator in the Mega element in *S*. *pneumonia*e with predicted terminators from mobile elements found in other Gram-positive bacteria species. The nucleotide sequence of the stem-loop portion (not including the poly-uracil tract) of the predicted Rho-independent transcriptional terminators are aligned and single nucleotide polymorphisms are shaded gray. The predicted free energy (ΔG) for each terminator is indicated The Rho-independent terminator structure from the Mega element is shown below in dot-bracket notation. Abbr., Mega (macrolide efflux genetic assembly), *S*. *pneumoniae* GA17457; *Tn*2009, *S*. *pneumoniae* GA08825 *Tn*916-*like* element (Genbank accession no. AILK01000006); *Tn*2010, *S*. *pneumoniae* GA47628 *Tn*916-like element (accession no. AILC01000010); 5612IQ, *S*. *pneumoniae* 5612IQ complex (accession no. AJ971089); *Sdys_eq*, *S*. *dysgalactiae* subspecies *equisimilis* G51 *mef(A)* element (accession no. AM168138); *Tn*1207.1, *S*. *pneumoniae* England^14^–1 phage-related element (accession no. AILI01000002); *Tn*1207.3, *S*. *pyogenes* 2812A phage-related element (accession no. AY657002); CkluΦ, *Clostridium kluyveri* DSM 555 phage-related element (accession no. CP000673); ΦMGAS10394, *S*. *pyogenes* MGAS10394 phage-related element (access no. CP000003); Cper_IFI, *C*. *perfringens* IFI *mef(A)* element (access no. EU553549); *S*. *pyogenes* MB56Spyo045 Φm46.1-like (access no. JF501521); ΦM46.1, *S*. *pyogenes* Φm46.1 (access no. FM864213).

Further, the sequences of the homologous Rho-independent terminator from these elements were analyzed. The sequence for the predicted terminators of Mega, and Mega carried on Tn*2009* and Tn*2010* were identical. Other elements displayed one to four SNPs in the predicted terminator (Fig. 8). The *mef(I)* elements from pneumococcus and *S*. *dysgalactiae* subspecies *equismilis* contained two SNPs (SNPs 1 and 2) (Fig. 8). However, these SNPs correlated to nucleotides of the *mef(*E) terminator that were predicted to be unpaired and to have minimal impact on the stability of the structure. The *mef(A)* elements and the *mef(I)* element from the *C*. *kluyverii* phage element contained SNP1 and SNP2, and also displayed SNP3 and/or SNP4 at nucleotides that were predicted to base pair in the *mef*(E) terminator (Fig. 8). Both SNP3 and SNP4 were adenine substitutions of uracil, thus disrupting an A-T base pair and thus were predicted to result in decreased stability of the structures compared to Mega (Fig. 8).

The overall sequence conservation of the predicted Rho-independent transcriptional terminators suggested similar mechanisms of regulation the *mef* and *mel(msr(D))* genes in each of these elements. In support of this observation, the erythromycin MIC of strain England^14^–9 containing *mef(A)* on Tn*1207.1*, was induced 8-fold by subinhibitory concentrations (1.0 μg ml^-1^) of erythromycin, increasing from 20 μg ml^-1^ in the absence of erythromycin to 320 μg ml^-1^ when induced. These data suggest that transcriptional attenuation is the mechanism of repression/induction of efflux-mediated macrolide resistance in Gram-positive bacteria regardless of the *mef* allele and its associated mobile element.

## Discussion

In this study the mechanism of repression and induction of the macrolide efflux operon *mef(E)/mel* encoded on the Mega mobile element in *S*. *pneumoniae* and other Gram-positive bacteria was defined. Like *erm(B)*, *mef(E)/mel* is induced by the 14- and 15-membered macrolides such as erythromycin and azithromycin, but not by 16-membered macrolides such as spiramycin and midecamycin [21]. This suggested that the two-systems were controlled by similar mechanisms, that is, attenuation.

Attenuation of inducible *erm* genes typically occurs at the level of translation (reviewed in [7]) and is characterized by the presence of one or two small (8–20 amino acids) leader peptides located on the *erm* transcript 5’ of the start codon encoding the structural Erm protein. These leader peptides toggle the folding of the *erm* transcript between two alternate secondary structures; the attenuator that blocks expression of *erm*, and the anti-attenuator that promotes expression. Ribosomes bound by an inducing macrolide pause at a programmed stall site on the leader peptide(s) and disrupt the attenuator, allowing formation of the anti-attenuator. Constitutively expressed *erm* genes typically have mutations in their 5’ regulatory regions which prevent formation of the attenuator structure [21]. In some *erm* macrolide-inducible systems, attenuation occurs at the level of transcription. This is similar to translation attenuation with the distinction that transcriptional attenuators form Rho-independent transcriptional terminators in non-inducing conditions leading to premature termination of transcription of the structural *erm* gene. Transcriptional attenuation has been observed for *erm-*encoded methylases of the *erm(D)* class, which includes *erm(D)*, *erm(K)* and *erm(J)* found in *Bacillus* species [15,16,22]. We have previously found that expression of *mef(E)/mel* is controlled at the level of transcription [4,8]. Thus, the hypothesis that *mef(E)/mel* was transcriptionally attenuated was explored.

The transcriptional start (+1) of the *mef(E)/mel* operon was mapped by 5′ RACE to a guanine nucleotide located 327 bp upstream of the *mef(E)* start codon. A consensus pneumococcal promoter sequence was identified upstream that included a pneumococcal extended-10 box and a-35 box. Deletion of the putative promoter abolished expression of a *mef(E)-lacZ* transcriptional fusion and rendered the mutant susceptible to erythromycin. Restoration of the mutated promoter to the wild type restored resistance to wild type levels confirming the location of promoter.

Analyses of the 327 bp 5′ regulatory region of *mef(E)* downstream of the promoter revealed a feature common to attenuators involved in the regulation of *erm*-type methylases, including a small *orf*, *mef(E)L*, encoding a small peptide (MTASMRLR) (Table 2) preceded by a consensus ribosomal binding site (RBS1). The molecular signals dictating programmed ribosome stalling involve sequence-dependent interaction between the nascent peptide, the peptidyl transferase center in the exit tunnel and the inducing macrolide molecule [23]. Macrolide-induced stalling at sites located on the leader peptides prevents the attenuator from forming in favor of the anti-attenuator to promote expression of the Erm methylase. The amino acid sequence of Mef(E)L was very similar to the leader peptide identified in other inducible macrolide systems, including Msr(A)L (MTASMRLK) encoded upstream of *msr(A) Staphylococcus* and the transcriptionally attenuated *erm(K)* (MTHAMRLRFPTLNQ) and *erm(D*) (MTHSMRLR) genes [15,16]. Significantly, the stall site on the *erm(K)* leader peptide *erm(K)L* is MRLR and the minimal Erm(K)L sequence that will allow induction of *erm(K)* is MTHAMRLR. These are nearly identical to the predicted sequence of the Mef(E)L peptide. We demonstrated that *mef(E)L* leader peptide was required for induced transcription of the *mef(E)/mel* operon and for efflux-mediated macrolide resistance. Translational inactivation by of *mef(E)L* ribosomal binding site by site directed mutagenesis and truncation of the protein resulted in macrolide susceptibility regardless of induction, consistent with the requirement for ribosomal stalling and anti-attenuation.

Transcriptional attenuators require one or more Rho-independent terminators 5’ of the induced structural gene. Stem-loop R5/R6 (Fig. 6) was identified as the terminator of the *mef(E)/mel* attenuator. It has a G+C rich stem, was predicted to be stable, and was immediately followed by a run of five uracil residues. Deletion of the structure significantly increased basal-level expression and resistance to erythromycin. Stem-loop R3/R4 was also required for the regulation of *mef(E)/mel* expression and we identified a pair of distal 17 base perfect inverted repeats in the *mef(E)* 5′ regulatory region separated by 174 nucleotides. The location of the inverted repeats within critical structures of the attenuator suggested involvement in the attenuation mechanism. Deletion of the first inverted repeat sequence, IR1, resulted in high-level expression, but abolished induction by erythromycin confirming a role in *mef(E)/mel* regulation.

The RNA-Seq data provided additional clarity and strong evidence for a transcriptional attenuation model and the induction of *mef(E)/mel* by anti-attenuation of transcription in the presence of inducing macrolides. In untreated and non-macrolide antibiotic-treated samples, the sequence reads mapped precisely to the transcriptional start site and terminated at the 3′ end of the Rho-independent terminator. In the macrolide-induced sample, the reads mapped to the start site and continued through *mef(E)* and *mel* structural genes. Our observations do suggest that control of *mef(E)/mel* is influenced by regulatory mechanisms in addition to transcriptional attenuation. We have previously reported that the antimicrobial peptide LL-37 induces *mef(E)* expression and increased resistance to erythromycin and that the mechanism of induction appeared to be distinct from that of macrolides [9]. Also, in some attenuator deletion mutants expression and resistance were still inducible by erythromycin indicating an additional level of control of the *mef(E)/mel* promoter. Further, multiple mechanisms have been reported controlling *erm* gene expression. In addition to attenuation, erythromycin-induced ribosomal stalling increases expression of *erm* genes by protecting transcripts from 5′-to-3′ nucleolytic degradation [24–26]. In preliminary experiments we have observed that in *mef*(E)/*mel* attenuator-deficient mutants, erythromycin resistance was reduced when *mef(E)L* was deleted as compared to when *mef(E)*L and the ribosome stall site were present. This suggests that erythromycin-mediated ribosome stalling may increase *mef*(E)/*mel* mRNA stability in a similar manner as reported for several *erm* genes. Another regulatory mechanism has recently been reported for *erm(C)* that involved macrolide-induced ribosomal frameshifting [27]. Frameshifting during translation of the leader peptide *erm(C)L* requires an telithromycin-bound ribosome and an *erm(C)L*”shift-prone” sequence which is essentially a run of four or more uracil or adenine residues. The ribosomal shift promotes translation of *erm(C)* by a mechanism that has yet to be determined. We have previously demonstrated that telithromycin induced *mef(E)/mel* expression and increased resistance to non-ketolide antibiotics [8]. The other potential regulatory mechanisms will be explored in future work.

There are other important differences between *erm* methylase and *mef(E)/mel* regulation suggesting that the systems are not completely analogous. We have shown previously that induction of *mef(E)/mel* by macrolides is dependent upon the identity of the amino sugar side chains of the macrolide ring [21]. Additionally, constitutively resistant (cMLS_B_ phenotype) clinical isolates of many species have been identified and are readily selected in the laboratory setting ([17,28,29]. To date, there have been no reports of constitutively expressed *mef*-mediated macrolide efflux in clinical isolates or generated *in vitro*. This may suggest a severe fitness cost to unregulated expression of *mef(E)/mel* in contrast to the *erm* methylases.

The comparisons of the 5′ region of *mef(E)/mel* of Mega to *mef*-containing elements from pneumococci and other Gram-positive species revealed conservation of the 327 bp regulatory region and the predicted secondary attenuator structures. These data suggest that *mef(E)*, *mef(A)* and *mef(I)* genes and the adjacent *msr* genes in pneumococci, other streptococci and other Gram-positive species, are likely responsive to macrolide induction and regulation by similar transcriptional attenuation. We verified that *mef(A)/msr(D)* carried on Tn1207.1 was induced by erythromycin. Thus, *mef(E)/mel* in *S*. *pneumoniae* is a model for inducible *mef*-mediated expression in a variety of Gram-positive pathogens.

## Supporting Information

S1 Table Nucleotide primers used in this study. (DOCX)

S2 Table Streptococcus pneumoniae isolates sequenced in this study. ^a^ strain collection. Abbr., GA EIP, Georgia Emerging Infections Program; PMEN Pneumococcal Molecular Epidemiology Network; EIP, Emerging Infections Program (Centers for Disease Control, Atlanta, GA); Jain, Shabnam Jain (Emory University,
^b^ Locale, geographic location of isolation
^c^ Source, biological source of isolation
^d^ ST, multilocus sequence type
^e^ CC, clonal complex
(XLSX)

## References

[1] Ross JI, Eady EA, Cove JH, Cunliffe WJ, Baumberg S, et al (1990) Inducible erythromycin resistance in staphylococci is encoded by a member of the ATP-binding transport super-gene family. Molecular Microbiology 4: 1207–1214. 2233255 10.1111/j.1365-2958.1990.tb00696.x

[2] Sutcliffe J, Tait-Kamradt A, Wondrack L (1996) *Streptococcus pneumoniae* and *Streptococcus pyogenes* resistant to macrolides but sensitive to clindamycin: a common resistance pattern mediated by an efflux system. Antimicrobial Agents and Chemotherapy 40: 1817–1824. 8843287 10.1128/aac.40.8.1817 PMC163423

[3] Gay K, Stephens DS (2001) Structure and dissemination of a chromosomal insertion element encoding macrolide efflux in *Streptococcus pneumoniae* . J Infect Dis 184: 56–65. 11398110 10.1086/321001

[4] Ambrose KD, Nisbet R, Stephens DS (2005) Macrolide efflux in *Streptococcus pneumoniae* is mediated by a dual efflux pump (*mel* and *mef*) and is erythromycin inducible. Antimicrobial Agents and Chemotherapy 49: 4203–4209. 16189099 10.1128/AAC.49.10.4203-4209.2005 PMC1251515

[5] Santagati M, Iannelli F, Oggioni MR, Stefani S, Pozzi G (2000) Characterization of a genetic element carrying the macrolide efflux gene *mef(A)* in *Streptococcus pneumoniae* . Antimicrobial Agents and Chemotherapy 44: 2585–2587. 10952626 10.1128/aac.44.9.2585-2587.2000 PMC90116

[6] Mingoia M, Vecchi M, Cochetti I, Tili E, Vitali LA, et al (2007) Composite Structure of *Streptococcus pneumoniae* Containing the Erythromycin Efflux Resistance Gene *mef(I)* and the Chloramphenicol Resistance Gene *catQ* . Antimicrob Agents Chemother 51: 3983–3987. 17709462 10.1128/AAC.00790-07 PMC2151433

[7] Chancey ST, Zahner D, Stephens DS (2012) Acquired inducible antimicrobial resistance in Gram-positive bacteria. Future Microbiol 7: 959–978. 10.2217/fmb.12.63 22913355 PMC3464494

[8] Chancey ST, Zhou X, Zähner D, Stephens DS (2011) Induction of Efflux-Mediated Macrolide Resistance in *Streptococcus pneumoniae* . Antimicrobial Agents and Chemotherapy 55: 3413–3422. 10.1128/AAC.00060-11 21537010 PMC3122420

[9] Zähner D, Zhou X, Chancey ST, Pohl J, Shafer WM, et al (2010) Human antimicrobial peptide LL-37 induces *mefE/mel*-mediated macrolide resistance in *Streptococcus pneumoniae* . Antimicrob Agents Chemother 54 3516–3519 10.1128/AAC.01756-09 20498319 PMC2916318

[10] Sabelnikov AG, Greenberg B, Lacks SA (1995) An Extended-10 Promoter Alone Directs Transcription of the *DpnI*I Operon of *Streptococcus pneumoniae* . Journal of Molecular Biology 250: 144–155. 7541838 10.1006/jmbi.1995.0366

[11] Lorenz R, Bernhart SH, Honer Zu Siederdissen C, Tafer H, Flamm C, et al (2011) ViennaRNA Package 2.0. Algorithms Mol Biol 6: 26 10.1186/1748-7188-6-26 22115189 PMC3319429

[12] Zuker M, Stiegler P (1981) Optimal computer folding of large RNA sequences using thermodynamic and auxiliary information. Nucl Acid Res 9: 133–148. 6163133 10.1093/nar/9.1.133 PMC326673

[13] McCaskill JS (1990) The equilibrium partition function and base pair binding probabilities for RNA secondary structures. Biopolymers 29: 1105–1119. 1695107 10.1002/bip.360290621

[14] Wilson KS, von Hippel PH (1995) Transcription termination at intrinsic terminators: the role of the RNA hairpin. Proceedings of the National Academy of Sciences 92: 8793–8797. 7568019 10.1073/pnas.92.19.8793 PMC41053

[15] Kwak JH, Choi EC, Weisblum B (1991) Transcriptional attenuation control of *ermK*, a macrolide-lincosamide-streptogramin B resistance determinant from *Bacillus licheniformis* . J Bacteriol 173: 4725–4735. 1713206 10.1128/jb.173.15.4725-4735.1991 PMC208150

[16] Choi SS, Kim SK, Oh TG, Choi EC (1997) Role of mRNA termination in regulation of *ermK* . Journal of Bacteriology 179: 2065–2067. 9068656 10.1128/jb.179.6.2065-2067.1997 PMC178934

[17] Min Y-H, Kwon A-R, Yoon J-M, Yoon E-J, Shim M-J, et al (2008) Molecular analysis of constitutive mutations in *ermB* and *ermA* selected *In Vitro* from inducibly MLS_B_-resistant enterococci. Archives of Pharmacal Research 31: 377–380. 10.1007/s12272-001-1167-8 18409053

[18] de Vries LE, Christensen H, Agerso Y (2012) The diversity of inducible and constitutively expressed erm(C) genes and association to different replicon types in staphylococci plasmids. Mob Genet Elements 2: 72–80. 22934240 10.4161/mge.20109 PMC3429524

[19] Drider D, DiChiara JM, Wei J, Sharp JS, Bechhofer DH (2002) Endonuclease cleavage of messenger RNA in *Bacillus subtilis* . Mol Microbiol 43: 1319–1329. 11918816 10.1046/j.1365-2958.2002.02830.x

[20] Sievers F, Wilm A, Dineen D, Gibson TJ, Karplus K, et al (2011) Fast, scalable generation of high-quality protein multiple sequence alignments using Clustal Omega. Molecular Systems Biology 7: 539 10.1038/msb.2011.75 21988835 PMC3261699

[21] Chancey ST, Zhou X, Stephens DS. The Macrolide Efflux System Encoded by *mefE/mel(msrA)* in *Streptococcus pneumoniae* Is Induced by 14- and 15-membered, but Not 16-membered Macrolides; 2008 6 8–12; Reykjavik, Iceland.

[22] Kim H-S, Choi E-C, Kim B-K (1993) A Macrolide—Lincosamide—Streptogramin B Resistance Determinant from *Bacillus anthracis* 590: Cloning and Expression of *ermJ* . Journal of General Microbiology 139: 601–607. 8473865 10.1099/00221287-139-3-601

[23] Vazquez-Laslop N, Thum C, Mankin AS (2008) Molecular mechanism of drug-dependent ribosome stalling. Molecular Cell 30: 190–202. 10.1016/j.molcel.2008.02.026 18439898

[24] Sandler P, Weisblum B (1989) Erythromycin-induced ribosome stall in the *ermA* leader: a barricade to 5′-to-3′ nucleolytic cleavage of the *ermA* transcript. J Bacteriol 171: 6680–6688. 2592348 10.1128/jb.171.12.6680-6688.1989 PMC210563

[25] Bechhofer DH, Zen KH (1989) Mechanism of erythromycin-induced *ermC* mRNA stability in *Bacillus subtilis* . J Bacteriol 171: 5803–5811. 2478520 10.1128/jb.171.11.5803-5811.1989 PMC210439

[26] Bechhofer DH, Dubnau D (1987) Induced mRNA stability in *Bacillus subtilis* . Proceedings of the National Academy of Sciences 84: 498–502. 3099297 10.1073/pnas.84.2.498 PMC304236

[27] Gupta P, Kannan K, Mankin AS, Vazquez-Laslop N (2013) Regulation of gene expression by macrolide-induced ribosomal frameshifting. Molecular Cell 52: 629–642. 10.1016/j.molcel.2013.10.013 24239289 PMC3874817

[28] Rosato A, Vicarini H, Leclercq R (1999) Inducible or constitutive expression of resistance in clinical isolates of streptococci and enterococci cross-resistant to erythromycin and lincomycin. J Antimicrob Chemother 43: 559–562. 10350387 10.1093/jac/43.4.559

[29] Malhotra-Kumar S, Mazzariol A, Van Heirstraeten L, Lammens C, de Rijk P, et al (2009) Unusual resistance patterns in macrolide-resistant *Streptococcus pyogenes* harbouring *erm(A)* . J Antimicrob Chemother 63: 42–46. 10.1093/jac/dkn432 18952616

[30] Farley M, Baughman W, Arnold K (2002) The Georgia Emerging Infections Program: monitoring trends in invasive pneumococcal disease. Journal of the Medical Association of Georgia 91: 20–23. 12189959

[31] McGee L, McDougal L, Zhou J, Spratt BG, Tenover FC, et al (2001) Nomenclature of Major Antimicrobial-Resistant Clones of *Streptococcus pneumoniae* Defined by the Pneumococcal Molecular Epidemiology Network. J Clin Microbiol 39: 2565–2571. 11427569 10.1128/JCM.39.7.2565-2571.2001 PMC88185

[32] Sharma D, Baughman W, Holst A, Thomas S, Jackson D, et al (2013) Pneumococcal carriage and invasive disease in children before introduction of the 13-valent conjugate vaccine: comparison with the era before 7-valent conjugate vaccine. Pediatr Infect Dis J 32: e45–53. 10.1097/INF.0b013e3182788fdd 23080290

[33] Halfmann A, Hakenbeck R, Bruckner R (2007) A new integrative reporter plasmid for *Streptococcus pneumoniae* . FEMS Microbiology Letters 268: 217–224. 17328748 10.1111/j.1574-6968.2006.00584.x

[34] Dunny GM, Lee LN, LeBlanc DJ (1991) Improved electroporation and cloning vector system for Gram+ bacteria. Applied and Environmental Microbiology 57: 1194–1201. 1905518 10.1128/aem.57.4.1194-1201.1991 PMC182867

[35] Frohman MA (1993) Rapid amplification of complementary DNA ends for generation of full-length complementary DNAs: thermal RACE. Methods Enzymol 218: 340–356. 7685466 10.1016/0076-6879(93)18026-9

[36] Miller JH (1972) Experiments in molecular genetics A laboratory manual and handbook for *Escherichia coli* and related bacteria. Cold Springs Harbor, New York: Cold Springs Harbor Laboratory Press.

[37] Edgar R, Domrachev M, Lash AE (2002) Gene Expression Omnibus: NCBI gene expression and hybridization array data repository. Nucleic Acids Res 30: 207–210. 11752295 10.1093/nar/30.1.207 PMC99122

[38] Santagati M, Iannelli F, Cascone C, Campanile F, Oggioni MR, et al (2003) The novel conjugative transposon Tn1207.3 carries the macrolide efflux gene *mef*(*A*) in *Streptococcus pyogenes* . Microb Drug Resist 9: 243–247. 12959402 10.1089/107662903322286445

[39] Hu P, Yang M, Zhang A, Wu J, Chen B, et al (2011) Complete genome sequence of *Streptococcus suis* serotype 14 strain JS14. J Bacteriol 193: 2375–2376. 10.1128/JB.00083-11 21398551 PMC3133069

[40] Hummel A, Holzapfel WH, Franz CM (2007) Characterisation and transfer of antibiotic resistance genes from enterococci isolated from food. Syst Appl Microbiol 30: 1–7. 16563685 10.1016/j.syapm.2006.02.004

[41] Kwon AR, Min YH, Yoon EJ, Kim JA, Shim MJ, et al (2006) ErmK leader peptide: amino acid sequence critical for induction by erythromycin. Arch Pharm Res 29: 1154–1157. 17225466 10.1007/BF02969307

[42] Gryczan T, Israeli-Reches M, Del Bue M, Dubnau D (1984) DNA sequence and regulation of *ermD*, a macrolide-lincosamide-streptogramin B resistance element from *Bacillus licheniformis* . Mol Gen Genet 194: 349–356. 6429477 10.1007/BF00425543

[43] Darty K, Denise A, Ponty Y (2009) VARNA: Interactive drawing and editing of the RNA secondary structure. Bioinformatics 25: 1974–1975. 10.1093/bioinformatics/btp250 19398448 PMC2712331

