# Supplementary material for: Transcriptional Attenuation Controls Macrolide Inducible Efflux and Resistance in Streptococcus pneumoniae and in Other Gram-Positive Bacteria Containing mef/mel(msr(D)) Elements
Source: PLoS One. 2015 Feb 19;10(2):e0116254. doi: 10.1371/journal.pone.0116254 (PMC4335068; doi:10.1371/journal.pone.0116254)
Supplement: S1 Table — (DOCX) [file pone.0116254.s001.docx]

**Table S1.** Oligonucleotide primers used in this study.

| **Name** | **Nucleotide Sequence** |
| --- | --- |
| SC143-NEST | 5’ TAACGAATTCTGCCCATATTGCATA 3’ |
| RACE-T-F | 5’ GCGAATTCCGGATCCGTTCAGCGCAGGGTC 3’ |
| SC199-NEST | 5’ATCGCAATTCGATGCCAGATTTAAAG 3’ |
| SC10-NEST | 5’ ACCAGAATTCACGGACAGCTATCGCAT 3’ |
| RACE-TT | 5’ GTTCAGCGCAGGGTCTTTTTTTTTTTTTT 3’ |
| 1-R | 5’ GAATTCGCAGGTACCGATGC 3’ |
| 1-726-F | 5’ GCATGCATCGGTACCTGCGAATTCATTCCATAACTTTTATATTGA 3’ |
| 1-796-F | 5’ GCATGCATCGGTACCTGCGAATTCAGGAGGAACCGAAACTATG 3’ |
| 1022-59-F | 5’ GCAGACCAAAAGCCACATTATTCAAAATAGAAATTCAAG 3’ |
| 1022-59-R | 5’ AATGTGGCTTTTGGTCTGCATACAT 3’ |
| 494-726-F | 5’ TAAGAGTAGACAAAAATTCCATAACTTTTATATTGA 3’ |
| 494-726-R | 5’ TATGGAATTTTTGTCTACTCTTAACCTT 3’ |
| 740-777-F | 5’ TCCATAACTTTTATTGATATAAACAAAGATGTAG 3’ |
| 740-777-R | 5’ TATATCAATAAAAGTTATGGAATGAGAC 3’ |
| 798-1086-F | 5’ ATAAACAAAGATGTATATGCAGGAGATAATATA 3’ |
| 798-1086-R | 5’ CCTGCATATACATCTTTGTTTATATCAA 3’ |
| bgaA_F1 | 5’ CCTTCTTAACGCCCCAAGTTCATC 3’ |
| bgaA_F2 | 5’ CTCACATGAACTACATGATGAACCC 3’ |
| bgaA_R1 | 5’ CTGCTGCTACTGCTGCTTGG 3’ |
| SC108-E-F | 5’ CATGCGGATCCGATAAACGAAAGCCTTAGAG 3’ |
| APH-3-E-R | 5’ ACATCGGTCGACGCTTGTAGTTAAA 3’ |
| APH-E-F | 5’ TCGTACGGTACCTTATATAGAAGAT 3’ |
| ME-AP-E-F | 5’ CGTACGGTACCATGGAAAAATACAAACCTTGGA 3’ |
| ME-R-E-R5 | 5’ ATGCACTCGAGCAATCACAGCACCCAATACG 3’ |
| ME-AP-E-R | 5’ TGAATGTCGACTATCTTCTTCCTAT 3’ |
| SC10 | 5’ ACTTGTCAATCACGGACAGC 3’ |
| SC83 | 5’ AATAGTCGACTTCGCACCATCACAGAAGC 3’ |
| SC85 | 5’ ATATCTAGAGTCTCACTGCACCAGAGG 3’ |
| SC108 | 5’CGATAAACGAAAGCCTTAGAGC 3’ |
| SC143 | 5’ ATATCTAGATTGCCCTGCCCATATTGC 3’ |
| SC199 | 5’ ATACTTCCAATTCGATGCCAG 3’ |
| SC323 | 5’ CGGTTGGAGGAACATCTTTGTTTATATCAATTTATAGTATAACAC 3’ |
| SC324 | 5’ TGTTCCTCCAACCGAAACTATGACAGCCTCAATG 3’ |
| SC325 | 5’ TGAGGCTGTTCATAGTTTCGGTTCCTCCTAC 3’ |
| SC326 | 5’ ATGAACAGCCTCAATGCGTTTAAGATAAGCTGGC 3’ |
| SC327 | 5’ TGAGGCTCACATAGTTTCGGTTCCTCCTAC 3’ |
| SC328 | 5’ ATGTGAGCCTCAATGCGTTTAAGATAAGCTGGC 3’ |
| SC329 | 5’ TTAGGCTGTCATAGTTTCGGTTCCTCCTAC 3’ |
| SC330 | 5’ ATGACAGCCTAAATGCGTTTAAGATAAGCTGGC 3’ |
| SC331 | 5’ TATGGCTGTCATAGTTTCGGTTCCTCCTAC 3’ |
| SC332 | 5’ ATGACAGCCATAATGCGTTTAAGATAAGCTGGC 3’ |
| Dmef-F | 5’ TATCGGTACCGCGGCCGCGAATTCAATCGGAAGTATCATGTCACTTGCT 3’ |
| Dmef-R | 5’CCGATTGAATTCGCGGCCGCGGTACCGATAATTAAATCGGCACCAATCA 3’ |
| Dmel-F | 5’ATATGGTACCGCGGCCGCGAATTCAGTAGTTTATGAAATTAGAGATAAG 3’ |
| Dmel-R | 5’ACTGTATTCGCGGCCGCGGTACCATATACTTCTAATTCATTTATATC 3’ |
| 4500-R | 5’ACGAATTCGGATCCGGGCAGCTACGCACACGCATTCA 3’ |
